# Supplementary figures and images for: Out of Africa: The genomic footprints of Vietnamese Robusta coffee
Source: PLoS One. 2025 May 28;20(5):e0324988. doi: 10.1371/journal.pone.0324988 (PMC12118859; doi:10.1371/journal.pone.0324988)

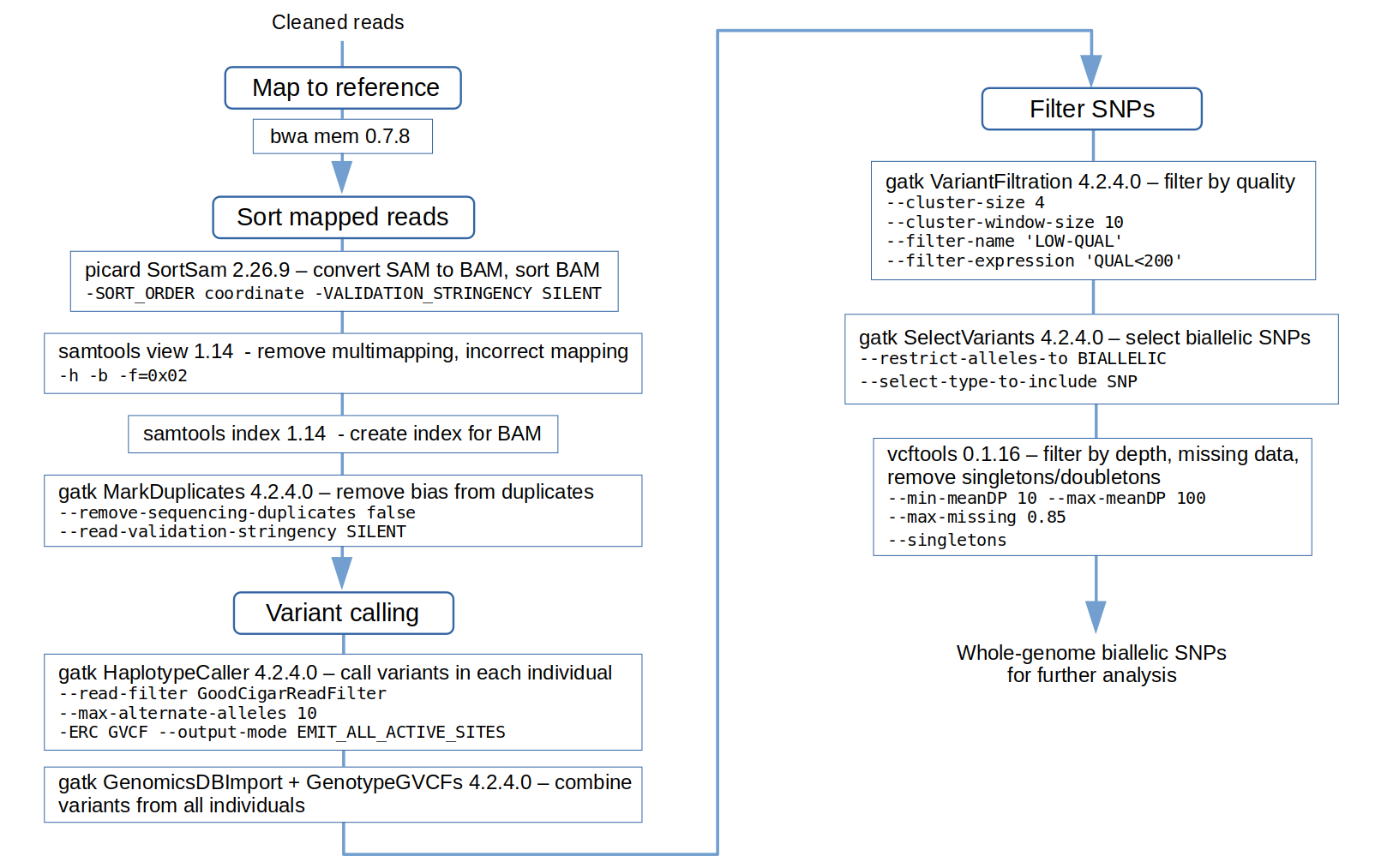

Supplement: S1 Fig — Software used: BWA mem 0.7.8, Picard Tools 2.26.9, SAMtools 1.14, GATK 4.2.4.0, vcftools 0.1.16. (PNG) [file pone.0324988.s006.png]

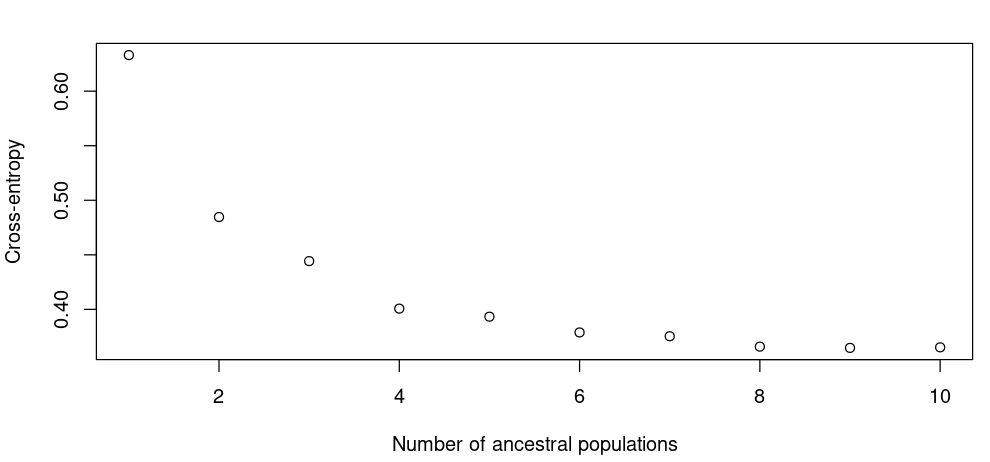

Supplement: S2 Fig — (PNG) [file pone.0324988.s007.png]

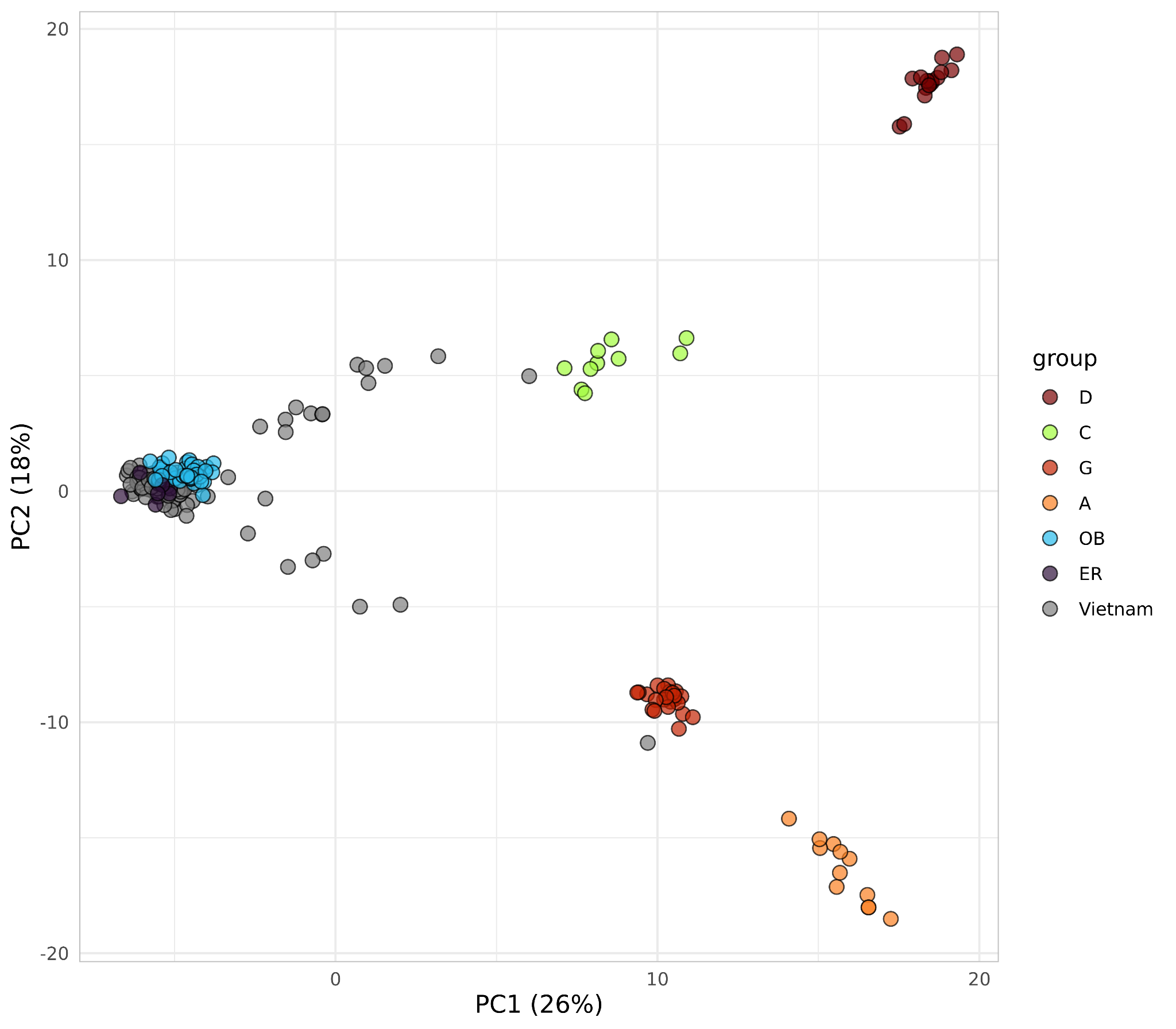

Supplement: S3 Fig — (PNG) [file pone.0324988.s008.png]

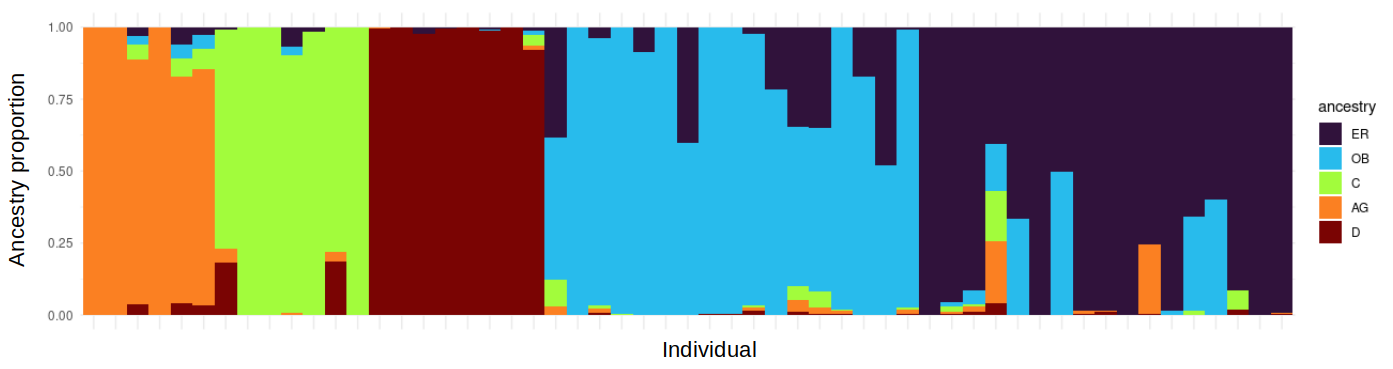

Supplement: S4 Fig — Five ancestral groups were estimated from the best sNMF run using a subset of 5 kb thinned SNPs. (PNG) [file pone.0324988.s009.png]

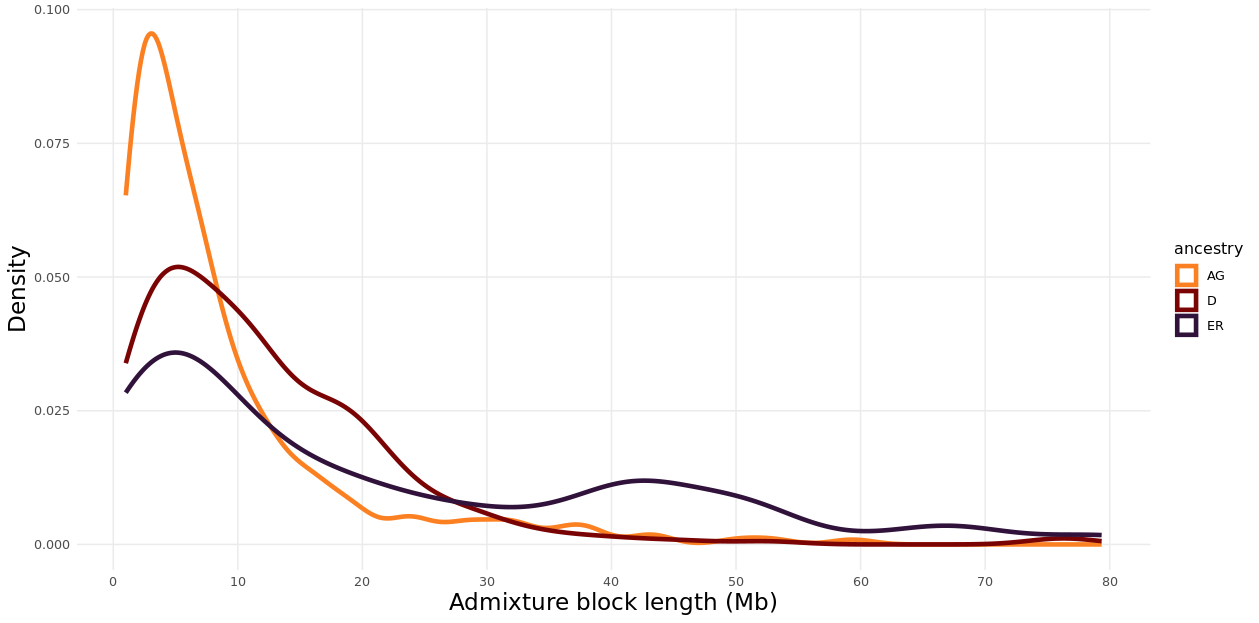

Supplement: S5 Fig — The admixture blocks were constituted of continuous admixture patterns. As the local ancestry inference was not based on phased sequencing data, the exact ancestry segments were unknown, but based on the parsimony principle, the probability of having 2 crossover events is higher than 3 or more events. The admixture blocks defined here were therefore with the most likely lengths. (PNG) [file pone.0324988.s010.png]

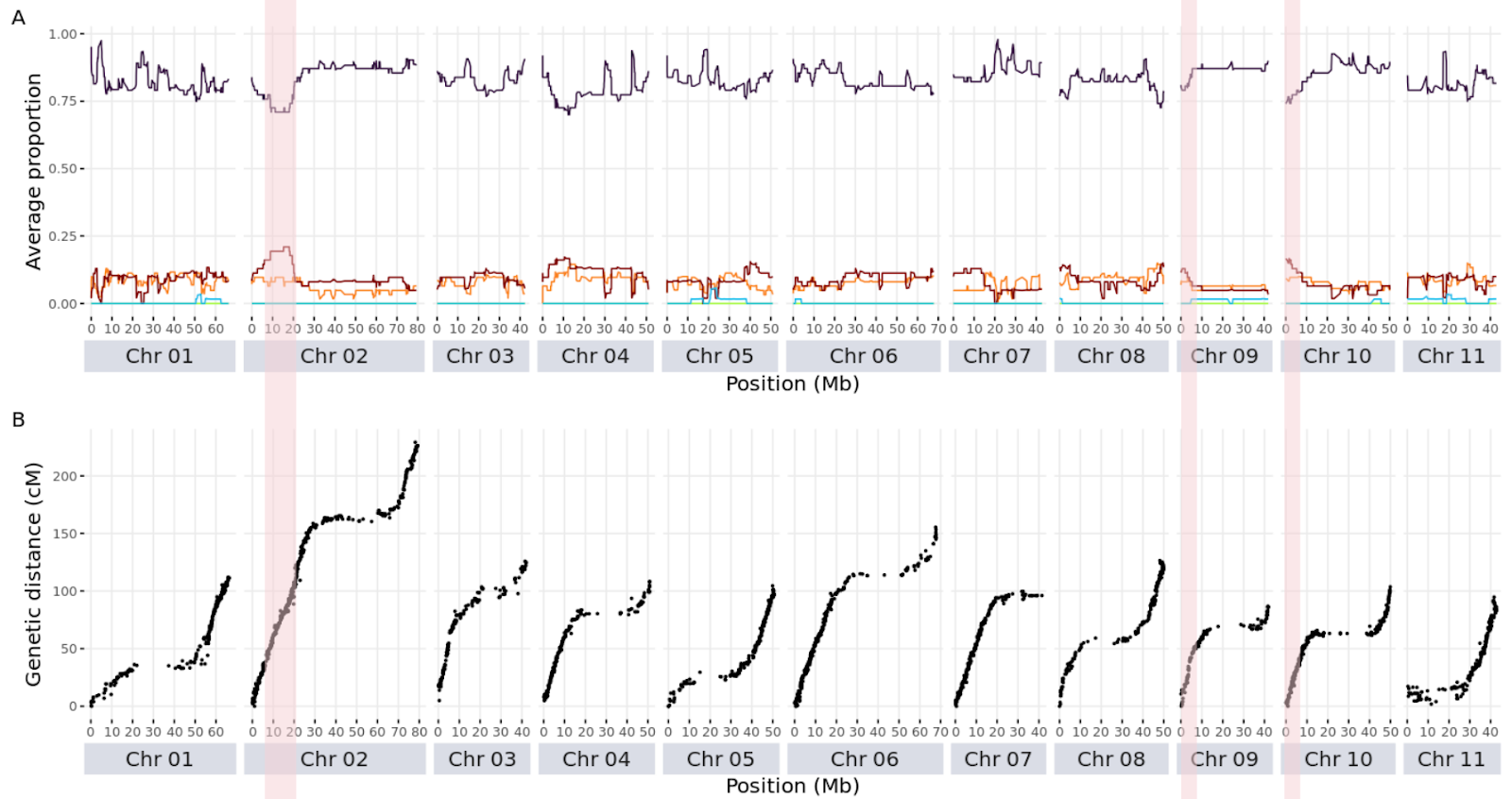

Supplement: S6 Fig — (A) Average proportion of the genetic groups at each SNP position. (B) Genetic distances along the chromosomes obtained from Mérot-Anthoëne et al. [7]. (PNG) [file pone.0324988.s011.png]
