# Supplementary material for: Out of Africa: The genomic footprints of Vietnamese Robusta coffee
Source: PLoS One. 2025 May 28;20(5):e0324988. doi: 10.1371/journal.pone.0324988 (PMC12118859; doi:10.1371/journal.pone.0324988)
Supplement: S1 Appendix — (PDF) [file pone.0324988.s012.pdf]

Appendix S1

Focus on the contribution of central Congolese (OB-ER) genetic groups to Vietnamese coffee trees

Analyses presented within the article revealed genetic contribution from Central Congolese Robusta to Vietnamese coffee. We sought to further investigate these origins by focusing on the Congolese genetic groups (O, B, E, R) and, furthermore, by adding new data from the INERA collections from the Democratic Republic of Congo to compare to Vietnamese coffee trees. These collections arose from Belgian colonial coffee breeding efforts at major historical centers of Robusta breeding: Lula, established ca. 1910-20, Yangambi, est. 1927 and the foremost breeding station in DRC and indeed Africa (and one of the major centers in the world) until the 1960s, and Luki [1,2].

To investigate the potential historical contribution of Robusta lines from these DRC collections to Vietnamese farms, we acquired additional KASP SNP genotyping data of several accessions held in the INERA collections [1], and additional wild reference accessions from previous work [3,4] (see table A1), encompassing many of the same target markers represented in the original data used for the main analyses in this study. Twelves samples were represented in both datasets and were used to confirm that they were in consensus. The Vietnamese, wild African accession and INERA collection samples data were then all combined, retaining only markers which were shared between them and showed 0% differences between repeat individuals—a total of 242—and individuals with <30% missing data.

Table A1: List of additional accessions included in the analyses presented in this appendix. Group assignment was based on a threshold of 70% contribution at K=6. Some samples are presumed to belong to the Congolese subgroup A but did not surpass the threshold in the present analysis. G0048, a wild specific from the Yangambi collection, was not assigned to a group by the population structure analysis under the retained threshold, but simple-matching distance calculated from genetic data suggests it may be closely related to, or part of, the OB group.

Accession label	Country	Population	sNMF group (K=6 on all samples)	Collection	Wild or Cultivar
BU-B-4	Uganda	Budongo	OB	NARO	wild
ZO-14	Uganda	Zoka	OB	NARO	wild
G0048	DRC	Bolondi		INERA Yangambi	wild
G0087	DRC	Luki (S19)	AG	INERA Yangambi	Luki cultivar. Presumed Congolese subgroup A
G0122	DRC	Lula	ER	INERA Yangambi	Yangambi cultivar. Presumed descendant Lula elite material
G0168	DRC	Lula	ER	INERA Yangambi	Yangambi cultivar. Presumed descendant Lula elite material
G0209	DRC	Luki (S19)		INERA Yangambi	Luki cultivar. Presumed Congolese subgroup A
G0210	DRC	Luki (LAF)		INERA Yangambi	Luki cultivar. Presumed Congolese subgroup A
G0223	DRC	Luki (PK)		INERA Yangambi	Luki cultivar. Presumed Congolese subgroup A
G0234	DRC	Lula	ER	INERA Yangambi	Yangambi cultivar. Presumed descendant Lula elite material

A new population structure analysis was performed using the sNMF function of the R package LEA [5] on this data, in the same manner as that presented in the main article, confirming both the results presented therein and the membership of three INERA reference accessions (from Lula lines) to the ER genetic group. We then created a "OB-ER" dataset (242 markers, 187 individuals) containing Vietnamese and reference accessions assigned to these Congolese genetic groups by using a threshold of 70% contribution from these groups at K=6.

Another population structure analysis was performed on this OB-ER dataset to reveal any potential structuring within the OB and ER genetic groups, with the same parameters. At K=4, the group OB and R were classified, and the group E was further differentiated into 2 subgroups - E_1 and E_2. The Vietnamese individuals in this dataset were clustered only in the OB or E subgroups (Fig A1).

A Ward's distance matrix was then calculated on the data using the gl.dist.ind function of the R package DartR [6] and used to perform a hierarchical clustering analysis using the hclust function in R. This analysis confirmed the INERA cultivars nestled within a clade containing elite and historical Vietnamese accessions, suggesting that the Yangambi station may indeed have been a source of material in the early development of Vietnamese coffee culture (Fig A1). The results also showed that several Vietnamese materials was obtained from the OB group populations.

Overall, these results point to the early history of coffee cultivation in Vietnam being more complicated than a single-source introduction, and highlight the possible diversity of genetic resources found in Vietnam (as well as in the WASI germplasm collection), even solely within the ER genetic group. Notably, several Vietnamese accessions belong to the OB subgroup, despite the absence of this group among the elite cultivars or the accessions identified as being older in Vietnam.

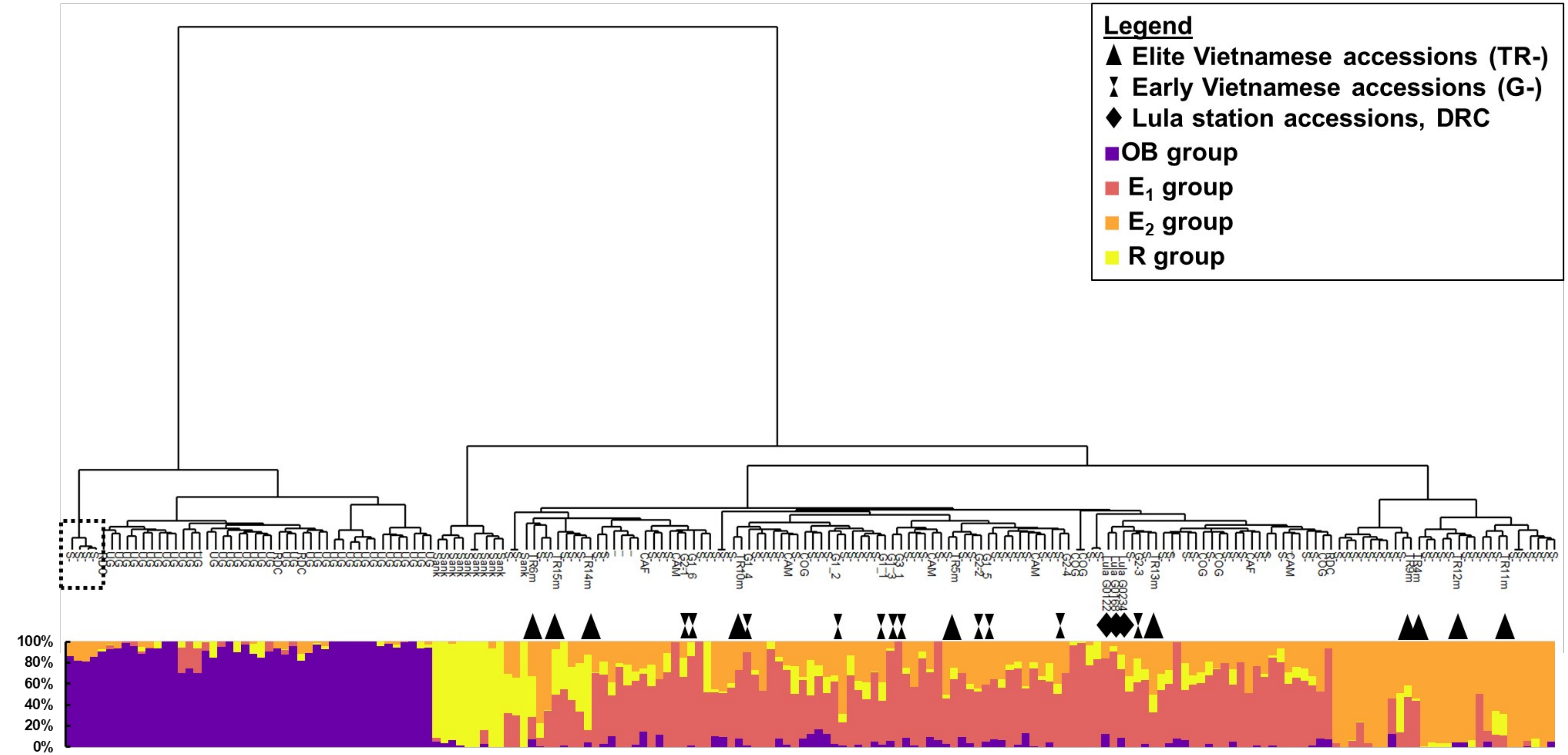

Figure A1: Structure of *Coffea canephora* OB and ER groups. Top: Hierarchical clustering dendrogram constructed from a Ward's distance matrix calculated on the OB-ER dataset of *C. canephora* from WASI and reference collections. Dashed line frame (**---**) in bottom left highlights Vietnamese farm accessions belonging to the OB group, of otherwise African accessions. Dendrogram leaf labels are as follows: TR*m: Vietnamese elite accessions, G*-*: early, historical Vietnamese accessions, S-: other Vietnamese accessions, CAF: Central African Republic accessions, CAM: Cameroon accessions, COG: Congo accessions, RDC: Democratic Republic of Congo accessions, UG: Ugandan accessions, Sank: R group accessions from Sankuru, Lula G0*: accessions from the Lula collection, INERA, DRC. The TR*m, G*-* and Lula G0* accessions are also highlighted with symbols on the figure. Bottom: Plot of sNMF population structure analysis (at K=4) performed on the OB-ER dataset, with sample order conserved from the dendrogram. The ER group was structured into R, E_1 and E_2.

References for this Supplementary notes

1. Verleysen L, Bollen R, Kambale J-L, Ebele T, Katshela BN, Depecker J, et al. (2023) Characterization of the genetic composition and establishment of a core collection for the INERA Robusta coffee (*Coffea canephora*) field genebank from the Democratic Republic of Congo. Frontiers in Sustainable Food Systems.;7: 1239442.

2. Montagnon C, Thierry Leroy, Albertus B. Eskes. (1998) Varietal improvement of *Coffea canephora*. 2 : Breeding programmes and their results. Plantations, Recherche, Développement, 1998. http://agritrop.cirad.fr/390311/.

3 Kiwuka, C., Goudsmit, E., Tournebize, R., Aquino, S. O., et al. (2021). Genetic diversity of native and cultivated Ugandan Robusta coffee (*Coffea canephora* Pierre ex A. Froehner): Climate influences, breeding potential and diversity conservation. PLoS ONE, 16(2), e0245965. https://doi.org/10.1371/journal.pone.0245965

4 Mérot-L'Anthoëne, V., Tournebize, R., Darracq, O., Rattina, V.,et al. (2019). Development and evaluation of a genome-wide Coffee 8.5K SNP array and its application for high-density genetic mapping and for investigating the origin of *Coffea arabica* L. Plant Biotechnology Journal, 17(7), 1418–1430. https://doi.org/10.1111/pbi.13066

5. Frichot E, François O. (2015) LEA: An R package for landscape and ecological association studies. O'Meara B, editor. Methods Ecol Evol.6: 925–929. doi:10.1111/2041-210X.12382

6. Gruber B, Unmack PJ, Berry OF, Georges A. (2018) DartR : An R package to facilitate analysis of SNP data generated from reduced representation genome sequencing. Molecular Ecology Resources.18: 691–699. doi:10.1111/1755-0998.12745
